# Inter-Trial Formant Variability in Speech Production Is Actively Controlled but Does Not Affect Subsequent Adaptation to a Predictable Formant Perturbation

**DOI:** 10.3389/fnhum.2022.890065

**Published:** 2022-07-07

**Authors:** Hantao Wang, Ludo Max

**Affiliations:** ^1^Department of Speech and Hearing Sciences, University of Washington, Seattle, WA, United States; ^2^Haskins Laboratories, New Haven, CT, United States

**Keywords:** speech motor control, variability, adaptation, auditory feedback, acoustics, articulation

## Abstract

Despite ample evidence that speech production is associated with extensive trial-to-trial variability, it remains unclear whether this variability represents merely unwanted system noise or an actively regulated mechanism that is fundamental for maintaining and adapting accurate speech movements. Recent work on upper limb movements suggest that inter-trial variability may be not only actively regulated based on sensory feedback, but also provide a type of workspace exploration that facilitates sensorimotor learning. We therefore investigated whether experimentally reducing or magnifying inter-trial formant variability in the real-time auditory feedback during speech production (a) leads to adjustments in formant production variability that compensate for the manipulation, (b) changes the temporal structure of formant adjustments across productions, and (c) enhances learning in a subsequent adaptation task in which a predictable formant-shift perturbation is applied to the feedback signal. Results show that subjects gradually increased formant variability in their productions when hearing auditory feedback with reduced variability, but subsequent formant-shift adaptation was not affected by either reducing or magnifying the perceived variability. Thus, findings provide evidence for speakers’ active control of inter-trial formant variability based on auditory feedback from previous trials, but–at least for the current short-term experimental manipulation of feedback variability–not for a role of this variability regulation mechanism in subsequent auditory-motor learning.

## Introduction

Over the years, the variability involved in human speech production has generated substantial empirical and theoretical interest. Both the physiological processes and acoustic output of speech production are inherently variable: even for a single speaker, no two repetitions of the same syllable are exactly the same in terms of muscle activation, kinematics, or acoustics (MacNeilage, 1970; Perkell and Klatt, 1986; Lindblom, 1990; Patri et al., 2015). Recently, it has started to become clear that such intra-individual variability at the behavioral level may reflect not only system noise but also functionally relevant adjustments in movement planning. Identifying the contribution of both these components will be critical for a better understanding of the sensorimotor control principles involved in spoken language.

To date, most experimental studies on the role of variability in speech production have taken an *observational* approach. That is, researchers typically have observed specific aspects of production variability in selected experimental conditions (without directly manipulating variability itself), and assessed the relationship with other measures of production or perception. For example, in the area of phonation, when subjects were asked to match a target tone by vocalizing with the same pitch and duration, those with greater production variability during the baseline phase exhibited stronger compensatory responses when unpredictable pitch perturbations were introduced in the auditory feedback signal (Scheerer and Jones, 2012). As an example from speech articulation, production variability for vowels has been shown to be linked to the speaker’s categorical perceptual boundary between vowels (Chao et al., 2019). Various studies also examined production variability in relation to aspects of perception, but quantified variability *across* different speaking conditions or consonant contexts (e.g., how different is /ε/ in “bed” vs. in “tech”), and thus did not address pure trial-to-trial variability in one particular phonetic context (e.g., Perkell et al., 2008; Franken et al., 2017).

Other groups have examined the potential relationship between observed trial-to-trial variability in speech acoustics and the extent of auditory-motor *learning* in a formant-shift adaptation paradigm. Purcell and Munhall (2006) reported a significant correlation between the lag 1 autocorrelation of trial-to-trial differences in a speaker’s first formant (F1) during a baseline phase with unaltered auditory feedback and the extent of subsequent adaptation in response to a F1 perturbation. The relevance of this report is unclear, however, as calculating the lag 1 autocorrelation based on *differences* between neighboring trials can be a form of overdifferencing (Cryer and Chan, 2008). For example, it can be mathematically demonstrated that, after differencing, even a white noise time series has a lag 1 autocorrelation of –0.5. Thus, finding a negative lag 1 autocorrelation based on differenced data does not necessarily mean that, in the original time series of formant data, trials were actually adjusted based on the preceding trial. In subsequent work, the same group quantified variability of vowel production as the standard deviations of a speaker’s F1 and F2 distributions during the baseline phase (MacDonald et al., 2011). Using pooled data from seven experiments with a total of 116 participants, they found no significant correlation between these different metrics of variability and the extent of adaptation to an F1 perturbation. In a more recent study, the same group did report a significant correlation between baseline F1 standard deviation and F1 adaptation, but they also cautioned–on the basis of a permutation test applied to the prior data–that this was most likely a chance result (Nault and Munhall, 2020).

Thus, the question whether individual speakers’ baseline formant variability relates to their extent of auditory-motor learning in a formant-shift adaptation task remains unanswered to date. Interestingly, a study on upper limb sensorimotor control has suggested that reach movement trial-to-trial variability during a baseline phase does, in fact, facilitate early learning when adapting to a perturbing force field, possibly because greater variability offers more exploration of the task space (Wu et al., 2014). Even for upper limb movements, however, the generalizability and interpretation of this single study remain unclear (He et al., 2016; Singh et al., 2016; Murillo et al., 2017; Sternad, 2018; van der Vliet et al., 2018).

A more powerful approach toward addressing the issue of a potential relationship between sensorimotor variability and sensorimotor learning may consist of investigating variability with *experimental*, rather than observational, research methods. Direct experimental manipulation of inter-trial motor and/or sensory variability would allow one to ask multiple more specific questions. First, is inter-trial variability itself under active control by the central nervous system? In other words, can we find evidence of adjustments that compensate for increases or decreases in perceived variability of a specific performance measure? Second, does either the change in perceived variability of a performance measure or any active motor compensation for that perceived change affect sensorimotor adaptation in a new environment where that same aspect of performance is predictably perturbed?

To start investigating speech variability with such experimental methods, it is possible to adapt an approach taken in upper limb studies that magnified or attenuated visual feedback errors by a certain ratio (van Beers, 2009; Wong et al., 2009; Patton et al., 2013; van der Kooij et al., 2015). By aiming to manipulate the magnitude of feedback error in each trial, those studies also magnified and/or attenuated the *dispersion* of feedback error across trials. Hence, similar manipulations can be used to answer the above formulated question whether the inter-trial variability for a particular parameter of motor performance is actively controlled by the central nervous system. Specifically, motor behavior can be analyzed for any evidence of adjustments that compensate for the magnified or attenuated feedback variability. It should be noted that, in this context, a study’s ability to both magnify and attenuate variability is critical from a methodological perspective. If an experimental paradigm only magnifies perceived variability by increasing the size of perceived movement errors, it is not possible to unambiguously attribute any resulting decrease in motor variability to the across-trials statistics *per se* vs. a preference for avoiding larger errors. If, on the other hand, a manipulation that attenuates perceived variability by minimizing perceived error leads to a compensatory *increase* in motor variability, then an interpretation based on the feedback statistics across trials is much more compelling as there are no theoretical reasons to expect a preference for avoiding smaller motor errors.

A reaching movement study by van Beers (2009) implemented such separate feedback conditions: movement endpoint errors were unaltered, reduced in magnitude by 50%, or increased in magnitude by 50%. Although the study did not specifically focus on compensation in terms of motor variability, van Beers (2009) found that the temporal structure of motor adjustments across trials differed among the visual feedback conditions: the sample lag 1 autocorrelation for movement endpoints was close to zero when errors in the feedback (and thus inter-trial variability) were not manipulated, negative when feedback errors were magnified, and positive when feedback errors were attenuated. The findings were interpreted in terms of which model of motor learning best explains subjects’ trial-to-trial adjustments, taking into account separate sources of central motor planning noise and peripheral motor execution noise. For natural movements with unperturbed feedback, van Beers (2009) concluded that trial-to-trial corrections are proportional to the magnitude of the previous error in such a way that movement variability is *minimized*, and it was suggested that this strategy is likely to underlie other forms of motor learning.

Lastly, a few upper limb studies have examined the effect of error magnification or attenuation on sensorimotor learning of a separate perturbation such as a visuomotor rotation. Results from those studies indicate that error magnification leads to more complete and faster adaptation whereas error attenuation has the opposite effect (Patton et al., 2013; van der Kooij et al., 2015). Despite this observed difference in adaptation, it has been argued that the adaptive learning mechanism itself, as quantified by a simple state-space model with the two parameters retention rate and error sensitivity, would remained unchanged between the different sensory feedback conditions (van der Kooij et al., 2015). However, other models of sensorimotor learning suggest that important parameters such as error sensitivity may be influenced by the prior history of feedback errors, a mechanism not captured by the simple state-space model (Herzfeld et al., 2014). Clearly, the effect of experimental manipulations of error magnitude and inter-trial variability on sensorimotor learning remains poorly understood even for upper limb movements.

Unfortunately, for speech articulation, work with experimental manipulations of feedback variability is only just starting to appear (see Tang et al., 2021), and the effect of manipulating the inter-trial variability of a specific parameter (e.g., frequency of one or more formants) on sensorimotor adaptation to a separate, predictable perturbation of the same parameter (e.g., a consistent formant shift) remains entirely unexplored. We therefore investigated whether an experimental magnification or attenuation of *perceived* inter-trial formant variability during speech production (a) leads to compensatory adjustments in *produced* formant variability, (b) induces changes in the temporal structure of formant adjustments across productions, and (c) affects subsequent auditory-motor learning when the speaker is exposed to a predictable formant-shift perturbation. Here, as the first step in this line of work, we implemented a relatively short-term formant variability manipulation (75 trials) and we looked for an effect on formant-shift adaptation in a subsequent task.

## Materials and Methods

### General Procedure

Twenty-eight right-handed adult native speakers of American English (20 women, 8 men, age *M* = 22.93 years, *SD* = 3.93years, range = 18–31) with no self-reported history of speech, hearing or neurological disorders participated after providing written informed consent (all procedures were approved by the Institutional Review Board at the University of Washington). Based on a pure tone hearing screening, all participants had monaural thresholds at or below 20 dB HL at all octave frequencies from 250 Hz to 4 kHz in both ears.

The experiment was conducted in a sound-attenuated booth. First, participants completed a practice session with unaltered auditory feedback to familiarize themselves with the instrumentation set-up by producing 7 blocks of three target words. Each block consisted of the monosyllabic words “talk,” “tech,” and “tuck” in randomized order. These words were presented individually on a monitor in front of the participant, each word remaining visible for 3 s. To help participants maintain a consistent speaking style, visual feedback about speech intensity and duration was presented on the monitor after each production. The target intensity was between 72 and 80 dB SPL, and the target vowel duration was 100–400 ms.

**FIGURE 1:**
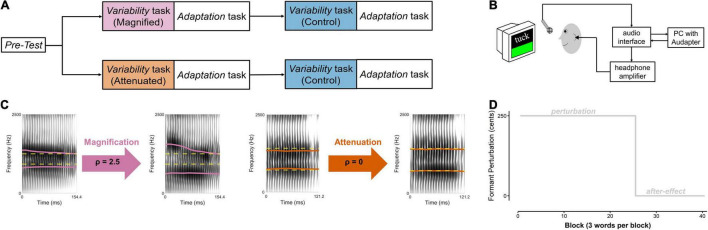
**(A)** Speech tasks completed by two groups of participants. Within each group, order of the experimental condition (Magnified or Attenuated feedback variability) and the Control condition was counterbalanced across participants. **(B)** Instrumentation setup. **(C)** Example spectrograms of Difference-shifted trials in the Magnified and Attenuated conditions of the *Variability* task. Dashed yellow line: pre-test median formant frequencies (F1 and F2, in Hz). Solid magenta and orange lines: produced (left of arrow) and heard (right of arrow) formants in the Magnified and Attenuated conditions. **(D)** Time course of the formant-shift feedback perturbation in the *Adaptation* task.

The actual experiment then included a *Pre-test* and two versions of a *Variability* task that were each immediately followed by an *Adaptation* task (Figure 1A). The *Pre-test* served to determine each participant’s median frequencies for the first and second formant (F1, F2) for the three target words (details below). During productions of the same words in the *Variability* tasks, inter-trial formant variability in the auditory feedback was either manipulated (*magnified* for one group of 4 men and 10 women, *attenuated* for the other group of 4 men and 10 women) or left unaltered (a control condition completed by both groups). Each *Variability* task was followed by an *Adaptation* task during which participants again produced the same target words but this time while hearing auditory feedback with a consistent upward perturbation of F1 and F2 (details below). The order of completing the manipulated and control versions of the *Variability* task (each immediately followed by an identical Adaptation task) was counterbalanced across participants.

In all of the above tasks, each participant’s speech output was captured with a microphone (SM 58, Shure) positioned 15 cm from the mouth and connected to an audio interface (Babyface Pro, RME, Haimhausen, Germany) and computer located outside the soundbooth (Figure 1B). The computer used MATLAB (The MathWorks, Natick, MA, United States) to present the visual stimuli, manipulate real-time auditory feedback when necessary, and record the participant’s speech. Auditory feedback manipulations were implemented with the publicly available MATLAB software ‘‘Audapter’’^1^ (Cai et al., 2008; Tourville et al., 2013). The output of the audio interface was amplified (HeadAmp6 Pro, ART ProAudio, Niagara Falls, NY, United States), and played back to the participant *via* insert earphones (ER-3A, Etymotic Research Inc., Grove Village, IL, United States). Before each participant’s experiment, the feedback system was calibrated such that speech input with an intensity of 75 dB SPL at the microphone resulted in 72 dB SPL output in the earphones (Cornelisse et al., 1991). For this calibration procedure, the intensity of the auditory feedback in the earphones was measured using a 2 cc coupler (Type 4946, Bruel & Kjaer Inc., Norcross, GA, United States) connected to a sound level meter (Type 2250A Hand Held Analyzer with Type 4947 ½″ Pressure Field Microphone, Bruel & Kjaer Inc., Norcross, GA, United States).

#### Pre-test

In the *Pre-test*, participants produced 30 blocks of the three target words with unaltered auditory feedback. During the production of each word, F1 and F2 were tracked by Audapter in real time. After the task was completed, a custom-written MATLAB script extracted the average F1 and F2 values (in Hz) across the middle portion of each production (defined as the window 40–60% into the vowel), calculated the across-trials median F1 and F2 for each of the participant’s vowels /ɔ/ (“talk”), /ε/ (“tech”) and /∧/ (“tuck”), and identified the actual production closest to the pair of F1 and F2 median values for each vowel (closeness was defined based on Euclidean distance in F1-F2 space). The mid-vowel F1 and F2 values from the participant’s three productions identified in this manner–productions hereafter referred to as the pre-test medians for each vowel–were used to determine the magnitude of the feedback variability manipulation in the *Variability* task. There was a short break (∼2 min) between the *Pre-test* and the first *Variability* task.

#### Variability Task

Participants performed the *Variability* task once with auditory feedback in which F1 and F2 variability was experimentally manipulated (either *magnified* or *attenuated*, depending on the participant’s group assignment) and once with unaltered auditory feedback as a control condition. In each *Variability* task, they produced 25 blocks of the three target words (for this first study with a variability perturbation, the number of trials was chosen based on published data regarding the number of trials that is sufficient for participants to reach maximum compensation in studies with other perturbations; see, for example, Kim et al., 2020a). In the *magnified* and *attenuated* conditions, formant variability in the auditory feedback was manipulated by modifying the difference between the formants in a given trial and the pre-test median for that vowel.

Specifically, a new mode of formant shifting, Difference-shift, was implemented by modifying Audapter’s source code. In the new Difference-shift mode, the user supplies a target frequency for each formant (*F^T^*) and a modification ratio (ρ). Within each frame, Audapter shifts the formant frequencies according to the equation *F^fb^* = *F^T^*+ρ×(*F^c^*−*F^T^*),where *F^fb^* is the formant frequency in the feedback and *F^c^* is the formant frequency of the current production (both in Hz). Thus, Difference-shift modifies the difference between the current formant value and the target frequency by the modification ratio. For example, if the user enters 550 Hz as the target frequency for F1 and ρ = 2.5, then for an actual F1 value of 600 Hz, the Difference-shift mode shifts the output F1 to 675 Hz (550 + 2.5 50). When ρ=1, the Difference-shift mode magnifies the difference between the produced formant value and the target frequency, whereas the difference is attenuated when ρ<1.

In both the Magnified and the Attenuated conditions, the pre-test median of F1 and F2 for each vowel was supplied as the target formant frequency *F^T^*. To magnify the difference between the current production and the target formant frequency, ρ was set to 2.5 in the Magnified condition. To minimize the difference between the current production and the target, ρ was set to 0 in the Attenuated condition. Examples of individual productions and the corresponding manipulated feedback for each condition are included in Figure 1C. Note that if ρ=0, the Difference-shift would theoretically always shift the formant frequency to the target frequency, regardless of the current production. However, due to the intrinsic limitations of real-time formant tracking and the digital filtering techniques used to alter the signal, the actual ratio between produced frequency and Difference-shifted output frequency is not always identical to the supplied modification ratio. Given this situation that, in reality, ρ=0 reduces (but does not completely eliminate) feedback variability, it was chosen as the preferred ratio for the Attenuated condition. The overall effectiveness of the feedback perturbation for magnifying and attenuating feedback formant variability is described below in the Section “Results.”

#### Adaptation Task

Each *Adaptation* task followed immediately after one of the *Variability* tasks, and was identical after the manipulated and control versions of the *Variability* task. In both cases, it consisted of a perturbation phase (25 blocks) and an after-effect phase (15 blocks) (Figure 1D). No variability manipulation was applied, but, at the start of the perturbation phase, a sudden 250 cents^2^ upshift of F1 and F2 was introduced by Audapter. This formant shift was turned off, and participants received unaltered auditory feedback, during the after-effect phase. There was a short break (∼2 min) between the end of the first *Adaptation* task and the beginning of the second *Variability* task.

### Data Extraction and Analysis

The speech signal from all tasks (*Pre-test* task, *Variability* tasks, and *Adaptation* tasks) was digitized by Audapter. Using a custom-written MATLAB script, we examined the production data from all tasks offline to exclude productions containing production errors (e.g., mispronunciations or yawning; 0.45% of productions were rejected for this reason), manually marked the onset and offset of the vowel in each production based on visual inspection of its waveform and spectrogram, and extracted the first two formant frequencies (F1 and F2) as tracked by the linear predictive coding algorithm implemented in Praat (Boersma, 2001). To disentangle feedforward adaptive learning vs. online feedback-driven corrections within trials, F1 and F2 formant values for each trial were extracted both across an initial portion of the vowel (5–30% into its total duration) and a middle portion of the vowel (40–60% into total duration). Additionally, to verify accuracy of the auditory feedback manipulation in the experimental conditions of the *Variability* task (Magnified and Attenuated variability), we extracted F1 and F2 also across the same middle portion of the vowel in the recorded feedback signal.

Statistical analyses for the *Variability* task and the *Adaptation* task made use of paired two-sample *t*-tests or, in a few cases, one-sample *t*-tests, with the significance level set at 0.05. When multiple statistical comparisons were carried out as one family of tests, *p*-values were adjusted with the Holm–Bonferroni method (Holm, 1979). Cohen’s *d* was used for effect size calculations (Cohen, 1988). All statistical tests were conducted in the R software (R Core Team, 2019).

#### Analysis of the Variability Task

Formant frequencies measured for the initial and middle portions of vowels from the *Variability* task were normalized by conversion from Hz to cents. The medians (F1 and F2) of each vowel from each subject’s pre-test productions, also measured offline across the initial and the middle portions separately, were chosen as the reference frequency for the conversion. Similarly, the formants measured from the middle portion of the vowel in the auditory feedback signal were also converted with reference to each subject’s pre-test median frequencies for the middle portion.

A primary focus of analysis for the *Variability* task was the participants’ production variability. To quantify this production variability with a measure directly related to the nature of the perturbation itself (i.e., distance to the pre-test median formants), we formulated a distance index (DI), $D\,I=\sqrt{F\,1^{2}+F\,2^{2}}$, where F1 and F2 are a trial’s formant frequencies already expressed in cents relative to the pre-test median. For each production, two DI’s, DI_initial_ and DI_mid_, were calculated with the formant values that had been extracted from the non-overlapping initial and middle portions of that trial’s vowel. For the auditory feedback signal, there was only one DI measurement per trial, DI_fb_, as formant frequencies had been extracted only for the middle portion of the vowel.

First, to verify the effectiveness of our formant feedback variability magnification and attenuation by the Difference-shift implementation in Audapter, the ratio between the average DI_fb_ and average DI_mid_ of each participant’s experimental *Variability* task was compared to the ideal ratio based on the perturbation algorithm (assuming perfect formant tracking and signal processing). Second, to examine the effect of feedback variability manipulation on production variability (Wong et al., 2009), we compared both DI_initial_ and DI_mid_ between the Control condition and the experimental (Magnified or Attenuated) conditions. To explore the possibility of gradual changes in production variability during the course of the *Variability* task, these variables were considered not only for the whole task (25 blocks of 3 trials each) but also block-by-block and stage-by-stage (with a stage operationally defined as a series of 5 consecutive blocks). Third, to examine possible online feedback-based corrections in response to the variability manipulations, we also calculated the within-trial difference between DI_initial_ and DI_mid_ [note that this approach shows similarities with the “centering” measure used in previous studies of online feedback corrections (Niziolek et al., 2013; Niziolek and Kiran, 2018), but differs from it in that our DI measures determine each trial’s distance to the median production from the *Pre-test* in cents rather than distance to the median production of the analyzed dataset itself in mels]. For each experimental condition (Magnified, Attenuated) and each control condition (completed by the Magnified and Attenuated groups separately), we used one-sample *t*-tests to determine whether the within trial changes in DI were statistically significantly different from zero (i.e., whether or not “centering” toward the pre-test median occurred). For each group separately, we then used paired *t*-tests to determine whether any within-trial changes differed between the experimental and control condition.

Although analogous to the nature of the variability perturbation itself, one potential problem with the DI-based analysis is that it is theoretically possible for a participant to increase or decrease the average distance between their trial formant frequencies and the pre-test medians without increasing the actual dispersion of these trials in two-dimensional (F1, F2) acoustic space. For example, although extremely unlikely for real speech, it is theoretically possible that the formants for all trials could be moved further away from the pre-test median (thereby increasing DI) but always to the same location in acoustic space. For this reason, we followed up on statistically significant DI effects by also determining for each participant the size of the area in acoustic space covered by the relevant productions (i.e., trials produced in the Control condition or in a given stage of the experimental conditions). The size of this area was determined by means of 95% confidence ellipses, calculated based on formant frequencies from the initial portion of the vowels.

A secondary focus of the *Variability* task was to investigate possible effects of the variability manipulations on the temporal structure of formant adjustments across trials. Consistent with the approach used in previous non-speech studies (van Beers, 2009; van der Vliet et al., 2018), we compared between Control and experimental conditions the sample lag 1 autocorrelation function, ACF(1), calculated for the sequence of averaged formant frequencies (i.e., mean of F1 and F2) obtained at the initial portion of the vowel in each trial. Formally, $A\,C\,F\,\left(1\right)=\frac{1}{N}\,\frac{\sum_{n=1}^{N-1}{}^{\left(F\,\left[n+1\right]-\bar{F}\right)\,\left(F\,\left[n\right]-\bar{F}\right)}}{\sum_{n=1}^{N}{\left(F\,\left[n\right]-\bar{F}\right)}^{2}}$, where *N* = 75, $F[n]=\;(F1_{initial}[n]\;+\;F2_{initial}[n])/2$, and $\bar{F}=\frac{1}{N}\,\sum_{n=1}^{N}F\,\left[n\right]$.

#### Analysis of the Adaptation Task

Given that adaptation refers to adjustments in movement planning based on prior experience (as opposed to online feedback-driven corrections), only the formant frequencies measured at the initial portion of the vowel were used for analysis of the *Adaptation* task. These formant frequencies were normalized to cents with reference to the median formants of each vowel in blocks 16–25 of the *Variability* task immediately prior to the onset of the *Adaptation* task. The frequencies of F1 and F2, in cents, were averaged for each trial as in several of our prior studies (e.g., Kim et al., 2020a; Shiller et al., 2020).

We compared three metrics between the perturbation phases from the Control and experimental conditions: early adaptation extent, early adaptation rate, and final adaptation extent. Early adaptation extent was calculated by determining the average formant frequency across the first 15 trials of the perturbation phase. Early adaptation rate was defined as the slope of a linear regression function based on the formant frequencies of the same 15 trials. Final adaptation extent was calculated by determining the average formant frequency across the last 15 trials of the perturbation phase of the task.

## Results

### Variability Task

#### Effectiveness of the Feedback Variability Manipulations

**FIGURE 2:**
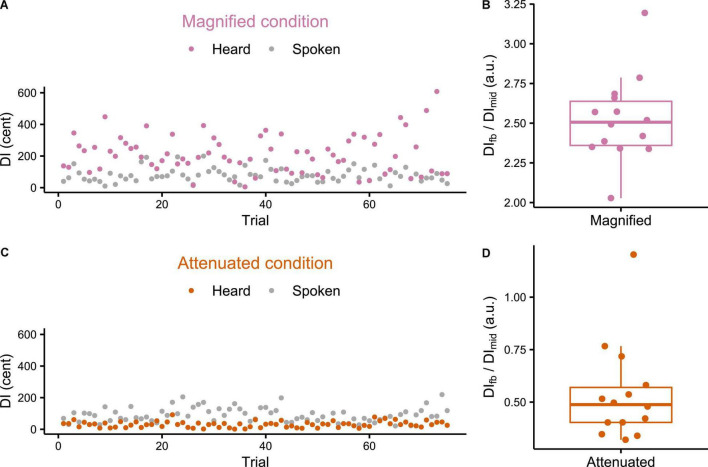
**(A,C)** Example individual participant data for production and feedback Distance Index (DI) of each trial in the *Variability* task under Magnified and Attenuated conditions. **(B,D)** Boxplots with symbols depicting each participant’s ratio between average feedback DI and average production DI (both measured mid-vowel) across all trials of the *Variability* task with Magnified or Attenuated feedback variability.

Individual participant data for DI calculated for both the produced and heard trials from the *Variability* task are presented in Figure 2. Figures 2A,C each show that the feedback manipulation was effective for the two selected participants from the Magnified and Attenuated conditions, respectively. Figures 2B,D show for *all* individual participants the ratio between the average DI of the formants in the manipulated feedback, DI_fb_, and that of the produced formants, DI_mid_, for the Magnified and Attenuated conditions, respectively. In the Magnified condition, the group mean of this ratio was 2.52 (*SD* = 0.27), a value very close to the intended modification ratio ρ=2.5 (which is also the theoretical value of DI_fb_/DI_mid_ if the Difference-shift had worked perfectly in every frame of every trial). In the Attenuated condition (ratio ρ=0), however, there was one outlier participant (DI_fb_/DI_prod_ = 1.202) for whom the Difference-shift mode failed to achieve the goal of attenuating formant variability in the auditory feedback. With the outlier removed, the group mean of the DI_fb_/DI_mid_ ratio was 0.49 (*SD* = 0.14) and all remaining ratios were less than 1, confirming that the goal of attenuating feedback variability was achieved. The data from the participant with the unsuccessful feedback perturbation in the Attenuated condition were excluded from all further analyses.

#### Production Variability

**FIGURE 3:**
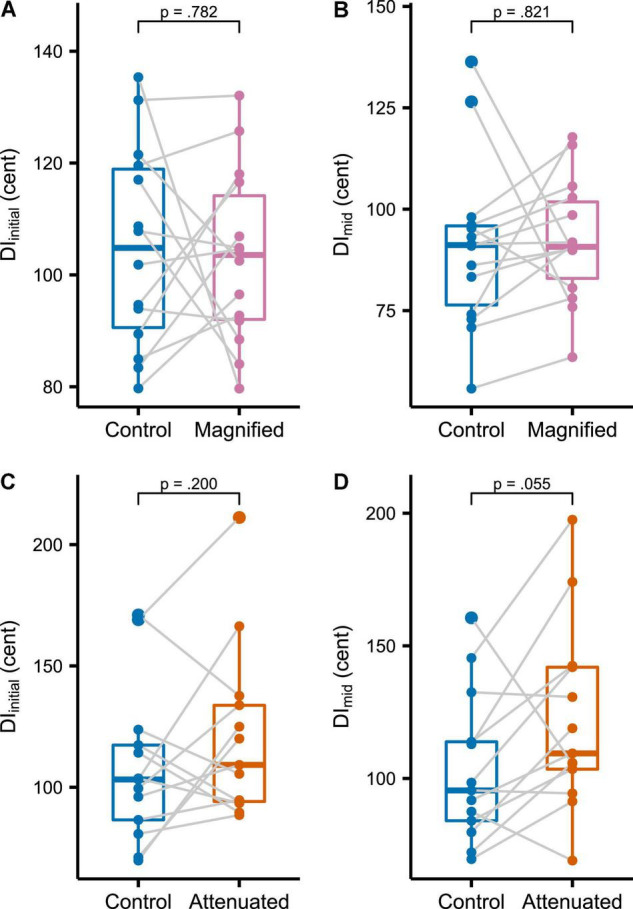
**(A,C)** Boxplots with symbols depicting each participant’s average DI_initial_ for the entire *Variability* task in Control and Magnified or Control and Attenuated conditions. **(B,D)** Boxplots with each participant’s average DI_mid_ for the entire *Variability* task in the same conditions.

The first set of production variability analyses compared the Control condition with both experimental conditions at the whole-task level for the target vowel’s initial portion (DI_initial_, Figure 3A for the Magnified condition, Figure 3C for the Attenuated condition) and middle portion (DI_mid_, Figures 3B,D). As compared with the Control condition, no statistically significant change in DI_initial_ was found for either the Magnified or the Attenuated condition [*t*(13) = –0.282, *p* = 0.782, *d* = 0.075, and *t*(12) = 1.358, *p* = 0.200, *d* = –0.376, respectively]. Similarly, there was also no significant change in DI_mid_ for either the Magnified or Attenuated condition [*t*(13) = 0.231, *p* = 0.821, *d* = –0.062, and *t*(12) = 2.122, *p* = 0.055, *d* = 0.588, respectively].

**FIGURE 4:**
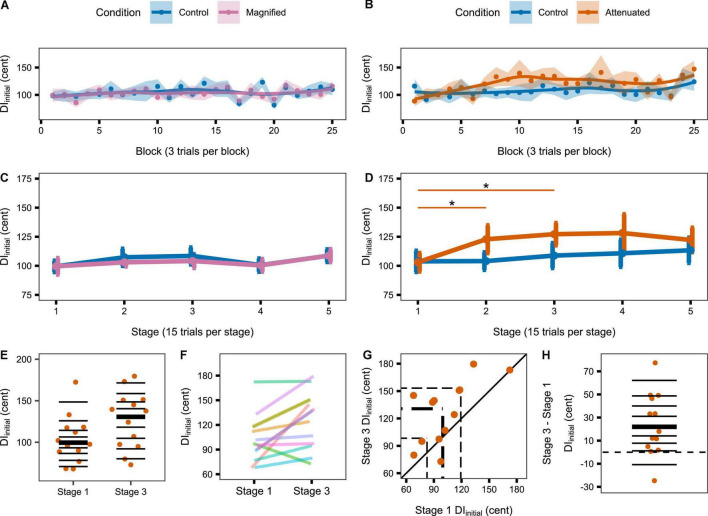
**(A,B)** Change in DI_initial_ across the Variability task by block (i.e., 3 trials) for the Magnified and Attenuated feedback variability conditions. Dots represent the group mean DI per block. Shaded regions indicate standard error of the mean (SEM). Solid lines are loess smoothed fits (span = 0.6). **(C,D)** Change in DI_initial_ across the Variability task by stage (i.e., 15 trials) for the Magnified and Attenuated feedback variability conditions. Error bars indicate SEM. Asterisks indicate adjusted p < 0.05 (see Table 1). **(E–H)** Individual participant data for the significant change from Stage 1 to Stage 3 in the Attenuated condition: **(E)** Stripchart of DI_initial_ in Stage 1 and Stage 3. Horizontal lines indicate deciles; bold line is the median. **(F)** Stripchart with each participant’s Stage 1 and Stage 3 data linked. **(G)** Scatterplot of Stage 1 by Stage 3 data. The diagonal line denotes no difference between stages. Participants in the upper left half increased DI_initial_ in Stage 3. Dashed lines mark quartiles. **(H)** Stripchart of the difference in DI_initial_ between Stage 3 and Stage 1. Horizontal lines indicate deciles; the bold line is the median; the dashed line is at zero (no difference between stages).

The second set of production variability analyses examined whether a response to the auditory feedback manipulations might develop over time with continuing exposure. Therefore, these analyses considered the time course of the DI_initial_ and DI_*mid*_ variables per block of 3 trials and per stage of 5 blocks. Figure 4 shows group data for the change in DI_initial_ from block to block (Figures 4A,B) and stage to stage (Figures 4C,D) under the Control and experimental conditions. For the group that completed Control and Magnified conditions, the data show no change in formant production DI_initial_ within either of those conditions. Statistical comparisons of DI_initial_ between the first stage and each of the following stages confirmed the absence of an adjustment in this distance metric with Magnified feedback variability (Table 1 and Figure 4C). In contrast, for the group that completed Control and Attenuated conditions, DI_initial_ showed a statistically significant increase from Stage 1 to Stage 2 and from Stage 1 to Stage 3 in the condition with Attenuated feedback variability whereas no statistically significant change was observed in the same group’s Control condition (Table 1 and Figure 4D). Visualizations of the Attenuated condition individual participant data for DI_initial_ in Stage 1 and Stage 3, and of the extent and direction for individual changes in this variable over the same time period, are included in Figures 4E–H (analysis techniques based on Wilcox and Erceg-Hurn, 2012; Bieniek et al., 2016; Rousselet et al., 2017). The data show a robust trend across individuals as 11 of 13 participants increased their formant production DI_initial_ in the first half of the task with Attenuated feedback variability.

**TABLE 1:** Adjusted *p*-values (paired *t*-tests, Holm–Bonferroni method) for comparisons of DI_initial_ (top section) and DI_mid_ (bottom section) between Stage 1 (first 5 blocks of 3 trials) and each subsequent stage (also 15 trials) in the Control, Magnified, and Attenuated feedback variability conditions (the two participant groups completing Magnified or Attenuated variability conditions each completed their own Control conditions, labeled Control M and Control A).

	Stage 1 vs. 2	Stage 1 vs. 3	Stage 1 vs. 4	Stage 1 vs. 5
	**DI_initial_**			
Control M	*t*(13) = –1.519, *p* = 0.612, *d* = –0.406	*t*(13) = –1.278, *p* = 0.612, *d* = –0.342	*t*(13) = –0.234, *p* = 0.819, *d* = –0.062	*t*(13) = –1.514, *p* = 0.612, *d* = –0.405
Magnified	*t*(13) = –0.506, *p* = 1.000, *d* = –0.135	*t*(13) = –0.680, *p* = 1.000, *d* = –0.182	*t*(13) = –0.111, *p* = 1.000, *d* = –0.030	*t*(13) = -1.717, *p* = 0.440, *d* = –0.459
Control A	*t*(12) = –0.041, *p* = 1.000, *d* = –0.011	*t*(12) = –0.561, *p* = 1.000, *d* = –0.156	*t*(12) = –1.153, *p* = 1.000, *d* = –0.320	*t*(12) = –0.944, *p* = 1.000, *d* = –0.262
Attenuated	*t*(12) = –2.800, *p* = 0.048*, *d* = –0.777	*t*(12) = –3.189, *p* = 0.031*, *d* = –0.884	*t*(12) = –2.330, *p* = 0.076, *d* = –0.646	*t*(12) = –2.051, *p* = 0.076, *d* = –0.569
	**DI_mid_**			
Control M	*t*(13) = –0.426, *p* = 1.000, *d* = –0.114	*t*(13) = –0.077, *p* = 1.000, *d* = –0.021	*t*(13) = –1.347, *p* = 0.804, *d* = –0.360	*t*(13) = –0.918, *p* = 1.000, *d* = –0.245
Magnified	*t*(13) = 0.473, *p* = 1.000, *d* = 0.126	*t*(13) = 0.676, *p* = 1.000, *d* = 0.181	*t*(13) = 0.501, *p* = 1.000, *d* = 0.134	*t*(13) = –2.526, *p* = 0.101, *d* = –0.675
Control A	*t*(12) = 0.659, *p* = 1.000, *d* = 0.183	*t*(12) = –0.126, *p* = 1.000, *d* = –0.035	*t*(12) = –1.133, *p* = 1.000, *d* = –0.314	*t*(12) = –0.427, *p* = 1.000, *d* = –0.118
Attenuated	*t*(12) = –3.039, *p* = 0.021*, *d* = –0.843	*t*(12) = –4.996, *p* = 0.001*, *d* = –1.386	*t*(12) = –1.929, *p* = 0.078, *d* = –0.535	*t*(12) = –3.406, *p* = 0.016*, *d* = –0.945

Statistically significant differences (*) were found only for the Attenuated feedback variability manipulation, in particular for the comparisons Stage 1 vs. Stage 2 and Stage 1 vs. Stage 3.

**FIGURE 5:**
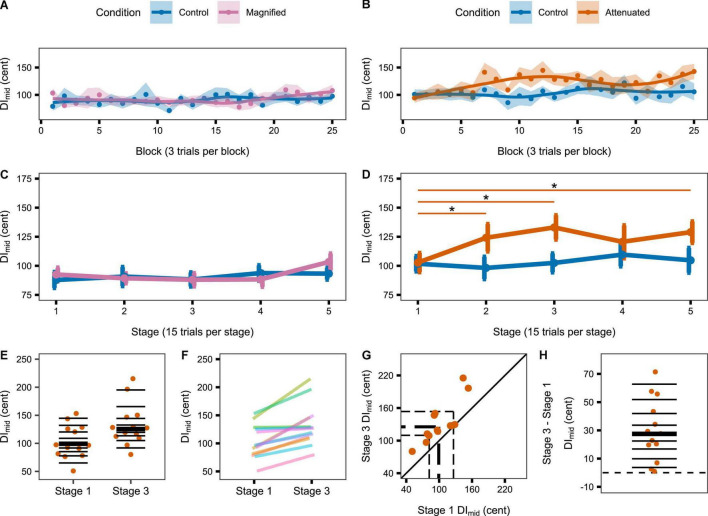
**(A,B)** Change in DI_mid_ across the Variability task by block (i.e., 3 trials) for the Magnified and Attenuated feedback variability conditions. Dots represent the group mean DI per block. Shaded regions indicate standard error of the mean (SEM). Solid lines are loess smoothed fits (span = 0.6). **(C,D)** Change in DI_mid_ across the Variability task by stage (i.e., 15 trials) for the Magnified and Attenuated feedback variability conditions. Error bars indicate SEM. Asterisks indicate adjusted p < 0.05 (see Table 1). **(E–H)** Individual participant data for the significant change from Stage 1 to Stage 3 in the Attenuated condition: **(E)** Stripchart of DI_mid_ in Stage 1 and Stage 3. Horizontal lines indicate deciles; bold line is the median. **(F)** Stripchart with each participant’s Stage 1 and Stage 3 data linked. **(G)** Scatterplot of Stage 1 by Stage 3 data. The diagonal line denotes no difference between stages. Participants in the upper left half increased DI_mid_ in Stage 3. Dashed lines mark quartiles. **(H)** Stripchart of the difference in DI_mid_ between Stage 3 and Stage 1. Horizontal lines indicate deciles; the bold line is the median; the dashed line is at zero (no difference between stages).

Similar results were obtained when considering DI_mid_ from block to block (Figures 5A,B) and stage to stage (Figures 5C,D): DI_mid_ showed no change in either group’s Control condition, also no change in the Magnified condition, but a statistically significant increase from Stage 1 to Stages 2, 3, and 5 in the Attenuated condition (Table 1 and Figure 5D). The individual participant data for Stage 1 and Stage 3 in this condition with Attenuated feedback variability show a highly consistent increase in formant production DI_mid_ during the first half of the task (Figures 5E–H).

We examined the change from DI_initial_ to DI_mid_ as an indicator of potential within-trial corrections in the conditions with Magnified or Attenuated formant variability in the auditory feedback. For participants assigned to the Attenuated group, within-trial changes were not statistically significantly different from zero for either the experimental condition [*t*(12) = 0.164, *p* = 0.872, *d* = 0.046] or the control condition [*t*(12) = –1.285, *p* = 0.446, *d* = –0.356]. For participants in the Magnified group, within-trial changes were statistically significant, but this was the case for both the experimental condition [*t*(13) = –4.117, *p* = 0.002, *d* = –1.100] and the control condition [*t*(13) = –3.650, *p* = 0.003, *d* = –0.975]. For neither group were within-trial changes in the experimental condition statistically different from those in the control condition with unaltered feedback variability [Attenuated group: *t*(12) = –0.997, *p* = 0.339, *d* = –0.288; Magnified group: *t*(13) = –0.962, *p* = 0.354, *d* = 0.264].

**FIGURE 6:**
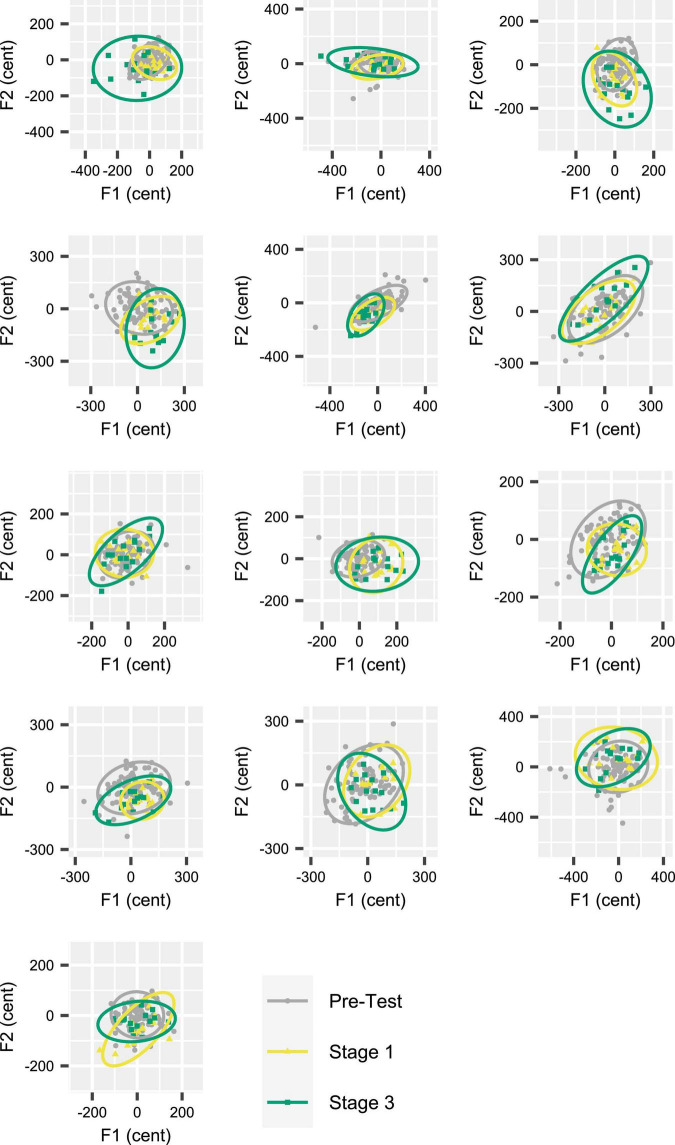
Individual participant data (one participant per panel) for inter-trial formant dispersion in acoustic vowel space (F1 by F2). Data based on 95% confidence ellipses, calculated for formant frequencies extracted from the initial portion of the vowels. Each participant’s data from Stages 1 and 3 (15 trials per stage) in the Attenuated feedback variability condition are shown together with their data from the *Pre-test* (90 trials). Nine of 13 participants increased ellipse area in Stage 3 as compared with Stage 1. Participants are ordered (by row) from greatest to smallest ellipse area increase.

Given that the Attenuated condition showed a statistically significant increase in DI_initial_ (as well as DI_mid_) from Stage 1 to Stage 3, Figure 6 shows the individual participants’ inter-trial dispersion of formant frequencies in 2D (F1, F2) acoustic space for Stages 1 and 3 of the Attenuated variability condition together with equivalent data from the *Pre-test*. All data were extracted from the initial portion of the vowels. Although the comparison of 95% confidence ellipse areas for Stage 1 versus Stage 3 did not reach statistical significance [*t*(12) = –1.894, *p* = 0.083], this comparison was associated with a medium effect size (*d* = –0.525), and 9 of 13 individual participants increased the ellipse area in Stage 3 as compared with Stage 1. Of the four participants who decreased ellipse size, only two showed a change that fell within the range of changes (but with opposite sign) observed for the subjects with increasing ellipses; the other two subjects showed only minimal changes.

#### Autocorrelation Structure

**FIGURE 7:**
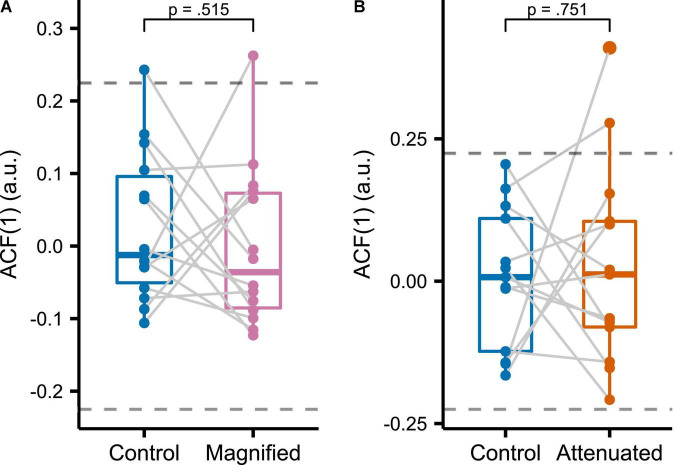
Sample lag 1 autocorrelation functions [ACF(1)] for formant data measured in the initial portion of the vowel and averaged across F1 and F2 for Control versus Magnified **(A)** and Control versus Attenuated **(B)** conditions of the *Variability* task. Dashed lines indicate the large sample 95% confidence interval of ACF(1) for a white noise process with sample size 75 (the number of trials per condition). Each dot represents an individual participant.

To assess the temporal structure of formant adjustments across the entire series of productions in the manipulated auditory feedback conditions, we determined the sample lag 1 autocorrelation [ACF(1)] of the time series consisting of averaged F1 and F2 values from the initial vowel portion of each trial in the *Variability* task (Figure 7). It should be noted that the large sample 95% confidence interval of ACF(1) for a white noise process with sample size *N* = 75 (i.e., the number of trials in each analyzed time series) is (-0.22, 0.22) (Brockwell and Davis, 2016). Most of the individual ACF(1) data from all conditions in the current study fell within this bound, indicating that, from a statistical perspective, it is likely that most production sequences were generated by white noise processes. There were no statistically significant differences in ACF(1) between either of the two experimental conditions and the Control condition [Magnified: *t*(13) = 0.670, *p* = 0.515, *d* = 0.179; Attenuated: *t*(12) = –0.324, *p* = 0.752, *d* = 0.090].

### Adaptation Task

**FIGURE 8:**
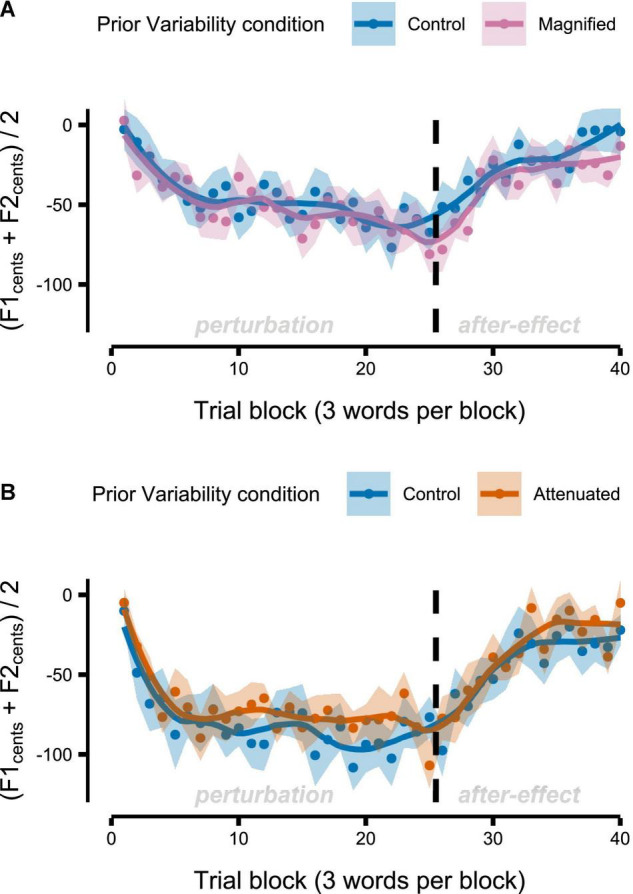
Group-level formant-shift adaptation data after completion of the *Variability* task’s Control and Magnified conditions **(A)** or after the Control and Attenuated conditions **(B)**. Dots represent group mean formant frequencies per block (3 trials) and averaged across F1 and F2. Shaded regions indicate standard error of the mean. Solid lines are loess smoothed fits (span = 0.3).

**FIGURE 9:**
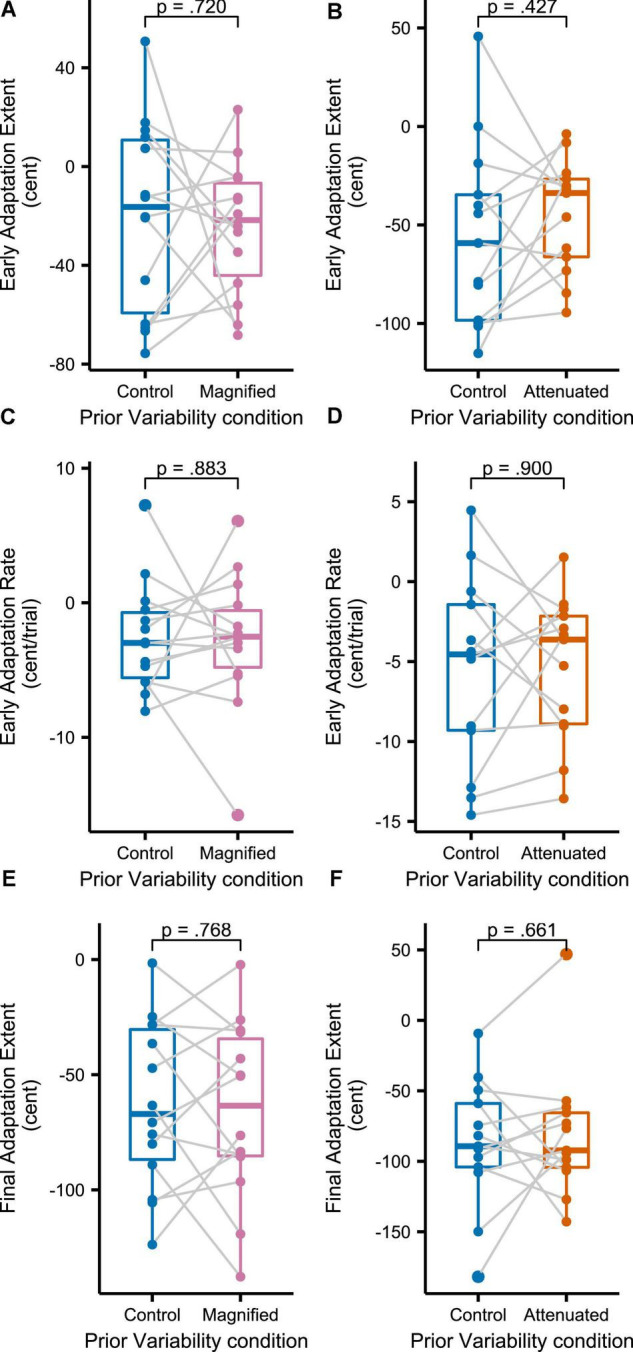
Boxplots with symbols depicting each participant’s early adaptation extent **(A,B)**, early adaptation rate **(C,D)**, and final adaptation extent **(E,F)** for formant-shift adaptation completed after the *Variability* task’s Control and Magnified or Control and Attenuated conditions. Full statistics for these data: **(A)** *t*(13) = 0.366, *p* = 0.720, *d* = 0.098; **(B)** *t*(12) = –0.822, *p* = 0.427, *d* = 0.228; **(C)** *t*(13) = 0.150, *p* = 0.883, *d* = 0.040; **(D)** *t*(13) = –0.128, *p* = 0.900, *d* = 0.035; **(E)** *t*(13) = 0.301, *p* = 0.768, *d* = 0.081; **(F)** *t*(12) = –0.450, *p* = 0.661, *d* = 0.125.

Figure 8 shows group mean formant frequencies produced throughout the *Adaptation* tasks that followed immediately after different conditions of the *Variability* task (data are in cents relative to the end of the preceding *Variability* task, measured at the initial portion of the vowel, and averaged across F1 and F2 and across the 3 trials per block). Recall that separate groups of participants completed the Magnified and Attenuated experimental conditions of the *Variability* task, and that, therefore, each group completed their own Control condition of the *Variability* task with no feedback perturbation. The Control versus experimental condition within-group comparisons in Figure 8 suggest that adaptation was not affected by the prior formant feedback variability manipulations. Statistical testing confirmed the absence of any significant differences between Control and Magnified or between Control and Attenuated for early adaptation extent (average formant frequency of the first 15 adaptation trials; Figure 9A), learning rate during early adaptation (slope of a linear regression line over the formant frequencies of the first 15 adaptation trials; Figure 8B), or final adaptation extent (average formant frequency of the last 15 perturbation trials; Figure 8C). The *p* values for all statistical comparisons are included with the data visualizations in Figure 9.

## Discussion

Previous observational studies have led to the suggestion that inter-trial motor variability may be related to both enhanced online feedback-based compensation (a study on fundamental frequency in speech, Scheerer and Jones, 2012) and enhanced adaptive learning (a study on upper limb reach movements, Wu et al., 2014). However, neither of these results have been consistently supported by other empirical data (Scheerer and Jones, 2012; He et al., 2016; Singh et al., 2016), alternative explanations for the findings have been offered (He et al., 2016; Singh et al., 2016; Murillo et al., 2017; Sternad, 2018; van der Vliet et al., 2018), and further investigation is clearly warranted (Dhawale et al., 2017). Moreover, results from an experimental study that directly manipulated feedback variability for reaching movements by magnifying or attenuating the size of target errors suggested that the temporal structure of adjustments across trials, indexed by the sample lag 1 autocorrelation [ACF(1)] for movement endpoints, changed with manipulated feedback (van Beers, 2009). In the same study, the adjustments across trials were consistent with predictions made by state-space models often used to characterize learning mechanisms in sensorimotor adaptation experiments (van Beers, 2009). Thus, inter-trial motor variability itself may represent a form of trial-by-trial learning. On the other hand, the authors of a reaching movement experiment combining error feedback magnification or attenuation with a constant perturbation that elicits visuomotor adaptation concluded that variability manipulation did not alter the underlying adaptive learning mechanisms (van der Kooij et al., 2015), despite observed changes in adaptation behavior (Patton et al., 2013; van der Kooij et al., 2015).

We sought to clarify, for sensorimotor control of speech articulation, whether *experimental manipulations* of inter-trial feedback variability (here variability of formant frequencies in the real-time auditory feedback) (a) lead to speaker adjustments in inter-trial production variability, suggestive of an active regulation mechanism; (b) lead to changes in the temporal structure of adjustments across trials [ACF(1)], suggestive of trial-by-trial learning; and (c) affect learning in a subsequent auditory-motor adaptation paradigm with a constant formant-shift perturbation. To manipulate inter-trial formant variability in the feedback, we implemented a novel real-time formant manipulation algorithm that can either magnify or attenuate the difference between the formants in a current production and target formants operationally defined as the median formant values from a *Pre-test*.

### Active Regulation of Variability

After the *Pre-test* with unaltered auditory feedback, participants completed two conditions of a *Variability* task (each followed by an *Adaptation* task): one was a Control condition with unaltered formant feedback, and the other condition had either Magnified or Attenuated formant variability in the auditory feedback, depending on the participant’s group assignment. Signal processing algorithms generating the feedback signal in these experimental conditions increased or decreased the distance between the formants produced in a given trial and the participants’ median formants for the same word in the *Pre-test*. We therefore quantified participants’ productions with a DI that expressed produced formant frequencies also in terms of their distance to the pre-test median.

Compared with each group’s own Control condition, the condition with Magnified feedback variability did not result in an adjustment in distance, but the condition with Attenuated feedback variability led to a gradual *increase* in distance between produced trials and the pre-test median (thus opposing the feedback manipulation). This increasing distance between produced formants and pre-test median formants was detected in both the initial portion of the vowel (5–30% into the total vowel duration; results in Figure 4) and the middle portion of the vowel (40–60% into the total vowel duration; results in Figure 5) portions of the vowel, and, thus, reflects gradual changes in movement planning rather than online within-vowel corrections. In fact, neither of the experimental conditions affected the extent of within-vowel corrections as compared with the same participants’ Control condition. As it is theoretically possible for DI to increase even in the absence of an increase in variability (e.g., if a participant moved their formants further from the pre-test median but always to the same location in acoustic F1F2 space), we followed up by determining the size of the area in acoustic space covered by each participants’ productions. This analysis confirmed that during the early stages of exposure to Attenuated variability feedback, most—but not all—participants did actually increase the overall spread of their productions in the two-dimensional acoustic space (i.e., increased formant production variability; results in Figure 6).

It is not straightforward to compare this finding of active variability regulation with those from prior limb motor control studies that magnified and/or attenuated the dispersion of feedback across trials as a by-product of manipulating the magnitude of target error in each trial. The study by Wong et al. (2009) only *increased* the size of perceived target errors (and thus feedback dispersion), and, consequently, one cannot necessarily attribute the resulting decrease in motor variability to the magnified feedback variability as opposed to a control strategy that seeks to avoid large errors on each trial individually. The study by van Beers (2009) did implement both magnified and attenuated target errors, but focused on the temporal structure of movement endpoint adjustments across trials (see below Section “Temporal Structure”). Nevertheless, for reaching movements with unperturbed visual feedback, van Beers (2009) reported that trial-to-trial adjustments are made in such a way that movement variability is minimized.

Our data from speech articulation are not consistent with the idea that the central nervous system generally aims to minimize variability. In fact, these data suggest a strikingly different situation: when the feedback perturbation magnified inter-trial formant variability, this extended variability was tolerated and not opposed, but when the perturbation attenuated inter-trial formant variability, articulation was gradually adjusted such that the acoustic output counteracted the perturbation. Thus, overall, the present data are consistent with the interpretation that a sufficiently large level of feedback variability is desirable, and that this level of variability is actively regulated through adjustments in motor planning.

In light of this overall support for the hypothesis that variability is actively regulated, it is reasonable to wonder why the increase in production variability in the Attenuated condition was not statistically significant in some of the later stages of the task. As shown in Figure 4, the increase in DI_initial_ relative to stage 1 was significant in stages 2 and 3 but not in stages 4 and 5 (in both cases *p* = 0.076 with medium effect sizes). Closer inspection reveals that, at Stage 4, the mean DI_initial_ value had further increased, but the standard error of the mean was also larger at this stage. At Stage 5, the mean DI_initial_ value did decrease, but it never returned to its original value from stage 1. As shown in Figure 5, the increase in DI_mid_ relative to stage 1 was still statistically significant in the last stage of the task, only not in the preceding Stage 4 (*p* = 0.078, medium effect size). Thus, there was a trend for the increased production variability to be not sustained at its maximum level in the later stages of the task, but any attempts at interpreting the specific results for Stage 4 would be purely speculative.

It should also be acknowledged that an alternative explanation might be offered for the absence of formant variability regulation in the Magnified condition of our *Variability* task. Specifically, one could argue that the highly practiced speech movements may have been performed with minimized variability from the very beginning of the task, and that, therefore, a floor effect prevented further reduction of this variability in the Magnified condition. This would be a reasonable argument as the lower bound of variability seems to be physiologically constrained by the stochastic nature of events in the peripheral motor system such as synaptic transmission (Calvin and Stevens, 1968) and muscle contraction (Clamann, 1969; Hamilton et al., 2004), which together are referred to as execution or performance noise in theoretical models of motor control (Van Beers et al., 2004; Cheng and Sabes, 2006; van Beers, 2009; Dhawale et al., 2017; van der Vliet et al., 2018). Only a separate component of motor variability, namely, planning or state noise (Cheng and Sabes, 2006; van Beers, 2009), may be subject to regulation by the central nervous system (Wu et al., 2014; Dhawale et al., 2017, 2019). Total system noise always comprises both execution and planning noise, and, thus, cannot be regulated to a level lower than that of the execution noise itself. In fact, work on limb motor control has estimated the planning noise to be substantially smaller than the execution noise, the former accounting for only about 20∼30% of total motor variability (Cheng and Sabes, 2007; van Beers, 2009; van der Vliet et al., 2018).

However, the argument that the central nervous system does control speech movements in such a way that total system noise is minimized is not compatible with our results from the Attenuated condition. There would be no reason to implement adjustments in the direction of *more* variability in this condition if the controller seeks to minimize total system noise (given that the Attenuated feedback signal indicates a variability level that is minimized even below the presumed lower bound in typical speech). Consequently, our results from the two conditions taken together support the aforementioned interpretation that, at least for speech articulation, a certain non-minimal level of feedback variability is desirable and actively maintained, possibly in function of providing sensorimotor exploration (Wu et al., 2014; Dhawale et al., 2017, 2019). Moreover, this conclusion implies that the speech motor control system not only calculates and keeps track of distribution features for key aspects of the auditory feedback signal (e.g., dispersion measures such as variance of the formant frequencies), but also compares these features with the expected distributions and then updates future movement planning accordingly (Parrell and Houde, 2019). If our findings are replicated in future studies, computational and conceptual models of speech motor control will need to start incorporating such more complex feedback mechanisms, analogous to suggestions that have been made in the non-speech motor control literature (e.g., Herzfeld et al., 2014; Dhawale et al., 2019).

### Temporal Structure

If articulatory adjustments in the Attenuated condition of the *Variability* task relied on error-based learning mechanisms similar to those driving auditory-motor adaptation with predictable formant perturbations (Houde and Jordan, 1998; Daliri and Dittman, 2019), then the temporal structure of adjustments across trials—such as indexed by the lag 1 autocorrelation [ACF(1)] of the overall sequence of productions—would be expected to vary depending on the feedback manipulation (van Beers, 2009). It should be noted at this time that the authors of one previous publication on variability in formant production suggested that their ACF(1) results indicated trial-to-trial adjustments even for speech produced without any auditory perturbation (Sitek et al., 2013). However, the lag 1 autocorrelation of –0.47 in that study was calculated based on *differences* between pairs of successive trials, thus introducing the problem of overdifferencing that we have discussed above in the Introduction (recall that after differencing even a white noise time series has a lag 1 autocorrelation of –0.5). With regard to the specific perturbation-related questions investigated in the present study, our results (illustrated in Figure 7) showed no statistically significant difference in ACF(1) for the sequences of trials produced in the conditions with Attenuated or Magnified formant feedback variability versus the Control condition with unaltered auditory feedback.

The lack of significant difference in ACF(1) between the Control and experimental conditions (Attenuated and Magnified) is not consistent with work by van Beers (2009). In the latter study, comparisons with a Control condition showed that ACF(1) decreased in a Magnified condition and increased in an Attenuated condition, in keeping with the prediction of a state-space model of adaptive learning based on sensory feedback (Cheng and Sabes, 2006). In fact, in our own study, most of the ACF(1) values for the sequences of productions fell within the 95% confidence interval of a white noise process, suggesting no feedback-based learning. One possible interpretation is of course that the speech control system simply does not modify productions based on auditory feedback from the immediately preceding trial. Although this control system clearly shows adaptation to predictable auditory perturbations (Houde and Jordan, 1998; Villacorta et al., 2007; Shiller et al., 2020), it is possible that such learning mechanisms are inactive in the absence of consistently maintained predictable perturbations (cf. Gonzalez Castro et al., 2014; Herzfeld et al., 2014). However, the statistically significant increase in formant production variability in the Attenuated condition does indicate a previously undocumented form of adaptive learning process during this *Variability* task.

We therefore speculate that the employed ACF(1) analysis may fail to capture the specific form of feedback-based learning in the *Variability* task. Several observations support this hypothesis. First, the state-space model of motor control predicts that when the parameter of error sensitivity (also known as adaptation rate) is very low, the ACF(1) of the trial sequence for each of the feedback manipulations implemented in the current experiment would be small, and the trial sequence would resemble a white noise process (van Beers, 2009; van der Vliet et al., 2018). It has been estimated recently that, in comparison with limb motor control studies which generally reported error sensitivity in the range of 30–50% (Baddeley et al., 2003; Cheng and Sabes, 2007; van Beers, 2009; van der Kooij et al., 2015), the error sensitivity for speech auditory-motor adaptation is, on average, as small as 4.8% (Daliri and Dittman, 2019). Second, it is known from previous studies that adaptive learning in speech production can differ between different vowels and words, and a given participant may even adapt for one vowel but follow the perturbation for another vowel (Houde and Jordan, 1998; Max and Maffett, 2015). In the current study’s *Variability* task, three different target words (“talk,” “tech,” “tuck”) were produced in pseudo-random order. Feedback-based learning under such circumstances may be very complex (e.g., How much does feedback from a trial of “tech” affect the production of “talk”? What is the influence of some trials being preceded by the same word and other trials by a different word?), especially if the history of feedback prior to the last trial is also taken into account (Herzfeld et al., 2014). Such complexity is not captured by the simple ACF(1) index. Third, the statistically significant change in formant production variability during the Attenuated condition of the *Variability* task indicates that the production sequence may be non-stationary, which renders ACF(1) difficult to interpret. Unfortunately, despite these various disadvantages of ACF(1), it is unclear which alternative measurements may be used to reveal the temporal structure of feedback-based adaptive learning in conditions with altered formant feedback variability.

### Effect of Variability on Adaptation

Immediately after having been exposed to Attenuated or Magnified formant feedback variability, participants completed a conventional speech auditory-motor adaptation task with a predictable upward shift of all formants. This *Adaptation* task allowed us to assess the potential effect of prior formant feedback variability on formant production learning. If sensorimotor learning is affected by the extent of perceived inter-trial variability (Herzfeld et al., 2014; Wu et al., 2014), then participants’ formant adaptation profiles can be expected to differ after experiencing Attenuated versus Magnified formant feedback variability. On the other hand, if inter-trial variability has no effect on the mechanisms underlying adaptive learning (van der Kooij et al., 2015), then participants’ formant adaptation profiles can be expected to remain unchanged between conditions.

Results shown in Figures 8, 9 indicate that three different measures of formant adaptation—early adaptation extent, early adaptation rate, and final adaptation extent—were all statistically indistinguishable between the Control condition and the two experimental conditions (Magnified or Attenuated feedback variability). In other words, the prior manipulation of formant feedback variability, or the participants’ motor adjustments to this manipulation, had no effect at all on the subsequent formant shift adaptation task. This result aligns with the conclusion of van der Kooij et al. (2015) who conducted a series of visuomotor rotation reach experiments with magnified or attenuated visual feedback errors. Although those authors observed behavioral differences across the feedback manipulation conditions, state-space model estimates of the underlying learning *mechanism* remained unchanged. In the current study, even the behavioral measures showed no differences at all in each group’s comparison of formant adaptation after the control versus experimental condition of the *Variability* task. Hence, our results for speech articulation suggest no direct relationship between formant variability perceived in a preceding task and the adaptive learning of formant output adjustments when subsequently exposed to a persistent formant perturbation.

The absence of an effect of formant feedback variability on formant production adaptation may relate to the aforementioned low error-sensitivity parameter in speech auditory-motor adaptation (Daliri and Dittman, 2019). Of course, it is also possible that this outcome is entirely specific to certain methodological aspects of our study. For example, we only implemented a relatively *short-term* feedback variability manipulation (75 trials), and examined formant-shift adaptation in a *subsequent* task. Future studies should also address the effect of longer-term variability manipulations and variability manipulations implemented during the auditory-motor adaptation task itself. Moreover, it might prove fruitful to develop methodological approaches that are able to dissociate the effects of manipulations that alter sensory variability (as implemented here) versus direct manipulations of motor variability (which alter both motor and sensory variability).

## Conclusion

In sum, by experimentally manipulating inter-trial formant variability in the auditory feedback signal for speech, the present study yielded three novel findings. First, formant production variability in speech production appears to be actively regulated to a desirable level rather than merely minimized. Second, under the conditions investigated here, the temporal structure of inter-trial formant changes was not affected by experimental manipulations of formant feedback variability. Third, for these specific test conditions, subsequent auditory-motor adaptation in a standard formant shift perturbation task was also not affected by the formant feedback manipulations. We hope that future empirical studies will be able to investigate the generalizability of these findings, and that future theoretical work will provide conceptual and computational accounts of the active regulation of inter-trial variability in the sensorimotor control of speech production.

## Data Availability Statement

The raw data supporting the conclusions of this article will be made available by the authors, without undue reservation.

## Ethics Statement

The studies involving human participants were reviewed and approved by University of Washington IRB. The participants provided their written informed consent to participate in this study.

## Author Contributions

HW collected and analyzed the data. Both authors designed the experiments and data analysis procedures, interpreted the data, wrote the manuscript, contributed to the article, and approved the submitted version.

## Author Disclaimer

The content is solely the responsibility of the authors and does not necessarily represent the official views of theNational Institute on Deafness and Other Communication Disorders or the National Institutes of Health.

## Conflict of Interest

The authors declare that the research was conducted in the absence of any commercial or financial relationships that could be construed as a potential conflict of interest.

## Publisher’s Note

All claims expressed in this article are solely those of the authors and do not necessarily represent those of their affiliated organizations, or those of the publisher, the editors and the reviewers. Any product that may be evaluated in this article, or claim that may be made by its manufacturer, is not guaranteed or endorsed by the publisher.

## Footnotes

1 Audapter settings for the present study were as follows: sampling rate 48,000 Hz, downsampling factor 3, nDelay factor 3, linear prediction model order 17 for male participants and 15 for female participants. The total feedback loop latency of the specific hardware and software setup is 11.37 milliseconds (Kim et al., 2020b). Given that Audapter detects vowel onsets and offsets based on a short-time root mean square (RMS) intensity threshold, we determined the optimal RMS threshold for vowel detection for each individual participant based on visual inspection of the RMS intensity contours of the last five trials from the practice/familiarization session.

2 The conversion formula between cents and Hz is: $F_{c\,e\,n\,t\,s}=1200\times \log_{2}\left(\frac{F_{H\,z}}{R_{H\,z}}\right)$, where *R*_*Hz*_ is a reference frequency. 100 cents = 1 semitone. For the perturbation in the *Adaptation* part, *F*_*cents*_=250, *R*_*Hz*_=*F^c^*, and *F*_*Hz*_=*F^fb^*. A 250 cents upshift approximately equals a 15.5% increase in Hz.
